# Crystal structure of the folded domains of Xrs2 from *Saccharomyces cerevisiae*

**DOI:** 10.1107/S2053230X25006867

**Published:** 2025-08-06

**Authors:** Ajeak Vigneswaran, Ke Shi, Hideki Aihara, Robert L. Evans, Michael P. Latham

**Affiliations:** a Department of Biochemistry, Molecular Biology and Biophysics University of Minnesota Minneapolis MN55455 USA; MAX IV Laboratory, Sweden

**Keywords:** Nbs1/Xrs2, DNA double-strand breaks, DNA damage repair

## Abstract

The MRE11–RAD50–NBS1/Xrs2 (MRN/X) protein complex acts as a first responder in DNA double-strand break repair and telomere-length maintenance, yet the structural architecture of the yeast ortholog Xrs2 has remained unresolved. In this study, we present the first structure of the folded N-terminal region of Xrs2 from *Saccharomyces cerevisiae*, resolved at 2.38 Å using X-ray crystallography. Like the previously determined crystal structures of *Schizosaccharomyces pombe* Nbs1, the folded structure of *S. cerevisiae* Xrs2 adopts an extended three-domain organization at its N-terminus. Electrostatic analysis reveals two distinct charged patches: a positively charged patch on the FHA domain and a negatively charged patch in the cleft between the FHA and BRCT1 domains. This charge segregation is likely to play a role in mediating interactions with various ligands.

## Abstract 2

We present the 2.4 Å resolution X-ray crystal structure of the N-terminal folded domains of *S. cerevisiae* Xrs2, confirming a tandem FHA–BRCT1–BRCT2 domain architecture similar to that of *Schizosaccharomyces pombe* Nbs1. Comparative structural analyses and electrostatic surface mapping suggest that the FHA and tandem BRCT domains serve as a key binding site for phosphorylated protein partners.

## 1. Introduction

The MRN/X complex is a multi-protein assembly composed of MRE11, RAD50 and NBS1 in higher eukaryotes or Xrs2 in certain yeast species and serves as an initial sensor and responder to DNA double-strand breaks (DSBs), which are among the most severe forms of DNA damage (Bunting & Nussenzweig, 2013 ▸; Romero-Laorden & Castro, 2017 ▸; Lengauer *et al.*, 1998 ▸; Wang *et al.*, 2004 ▸). In addition to its critical role in detecting, processing and signaling DNA DSBs to initiate the repair process, the MRN/X complex also has an indispensable function in preserving telomere integrity, thereby maintaining genomic stability and preventing premature cellular aging or genome instability (Lamarche *et al.*, 2010 ▸). Mutation of the essential MRN is implicated in the development of prostate, ovarian and breast cancer and can be an underlying cause of diseases such as Nijmegen breakage syndrome (Chrzanowska *et al.*, 2012 ▸; Seemanová *et al.*, 2006 ▸; Carney *et al.*, 1998 ▸) and ataxia–telangiectasia-like disorders (Rahman *et al.*, 2020 ▸; Heikkinen, 2005 ▸).

**Figure 1:**
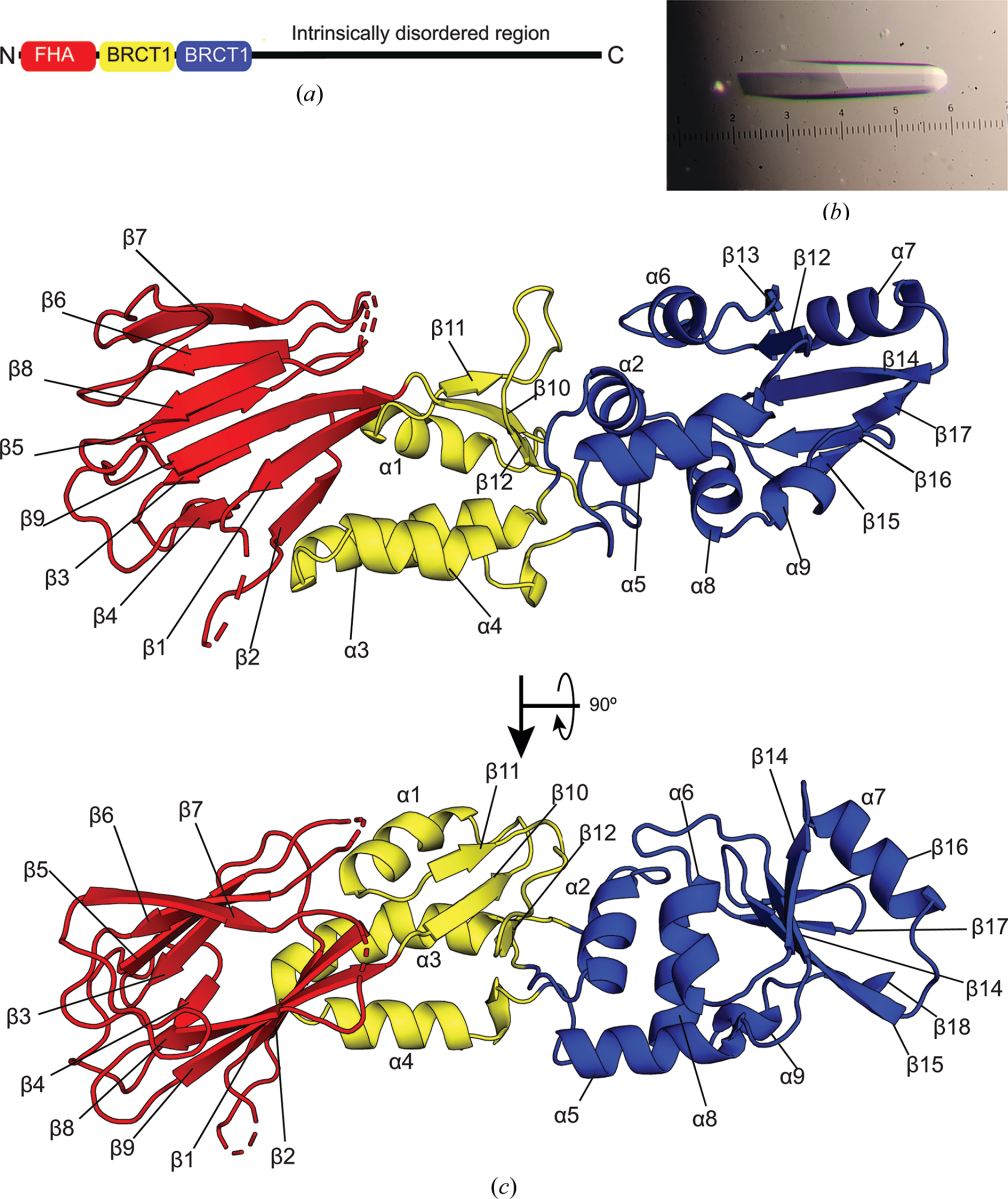
Structure of the *Sc* Xrs2^325^ FHA–BRCT1–BRCT2 region. (*a*) Cartoon representation of the *Sc* Xrs2 domain architecture. (*b*) The crystal of Xrs2^325^. (*c*) Ribbon representation of the *Sc* Xrs2^325^ crystal structure with major secondary elements (α-helices and β-strands) labeled. The FHA, BRCT1 and BRCT2 domains are colored red, yellow and blue, respectively, as in (*a*). All structural representations were made with *PyMOL*.

The universally conserved MRE11 and RAD50 subunits possess Mn^2+^-dependent nuclease and ATP-hydrolysis activities, respectively (Han & Huang, 2020 ▸; Lieber, 2010 ▸). NBS1/Xrs2 is a scaffolding hub for protein and DNA interactions with the complex (Kim *et al.*, 2017 ▸; Wu *et al.*, 2000 ▸; Oh *et al.*, 2016 ▸; Lu *et al.*, 2012 ▸; Schiller *et al.*, 2012 ▸; Trujillo *et al.*, 2003 ▸). NBS1/Xrs2 contains a folded region at the N-terminus and a long intrinsically disordered region (IDR) at the C-terminus (Fig. 1 ▸*a*). This folded region contains a forkhead-associated (FHA) domain (Durocher *et al.*, 1999 ▸) followed by tandem BRCA1 C-terminal (BRCT) domains (BRCT1 and BRCT2; Becker *et al.*, 2006 ▸). Phosphothreonine- and phosphoserine-binding sites in the FHA and BRCT1/2 domains, which are responsible for binding to phosphorylated histone γH2AX (Kobayashi *et al.*, 2002 ▸), CtIP/Sae2 (Williams *et al.*, 2009 ▸) and MDC1 (Kim *et al.*, 2006 ▸), alongside interaction motifs for RAD18, MRE11, RNF20, ATM/Tel1, phosphorylation and ubiquitylation sites and a nuclear localization signal (NLS) within the IDR, provide numerous protein-interaction sites on NBS1/Xrs2 (Williams *et al.*, 2009 ▸; Wu *et al.*, 2000 ▸; Oh *et al.*, 2016 ▸; Lu *et al.*, 2012 ▸; Schiller *et al.*, 2012 ▸; Kim *et al.*, 2017 ▸; Lloyd *et al.*, 2009 ▸; Kim *et al.*, 2006 ▸; Wu *et al.*, 2012 ▸; Kobayashi *et al.*, 2002 ▸). These protein–protein interactions are essential for localizing the MRN/X complex to the nuclease and bringing downstream effector proteins to the site of DNA DSBs. It has been proposed that the folded region of NBS1/Xrs2 can engage with other proteins through four potential binding modes: a binding partner can bind exclusively to either the FHA or BRCT1/2 domains, two different partners can bind separately to the FHA and BRCT1/2 domains, or a single partner can bind to both domains simultaneously (Williams *et al.*, 2009 ▸). For example, Tainer and coworkers have shown that *Schizosaccharomyces pombe* (*Sp*) Nbs1 binds a phosphothreonine peptide derived from Ctp1 (a homolog of Sae2 in yeast and CtIP in humans) through the FHA domain (Williams *et al.*, 2009 ▸). Additionally, two groups have demonstrated that *Saccharomyces cerevisiae* (*Sc*) Xrs2 binds directly to DNA, although it is unknown where this interaction occurs (Trujillo *et al.*, 2003 ▸; Möller *et al.*, 2024 ▸). These observations highlight the crucial role of NBS1/Xrs2 as a scaffolding hub for protein–protein and protein–DNA interactions.

Two X-ray crystal structures of *Sp* Nbs1 have been determined. Despite being orthologs, *Sp* Nbs1 and *Sc* Xrs2 display very low sequence similarity (∼30%), raising intriguing questions about how structural and functional conservation is maintained across such divergent sequences. Additionally, Xrs2, a core component of the yeast MRX complex, mirrors the role of human NBS1 in the MRN complex (both proteins bind and localize CtIP/Sae2 and ATM/Tel1 to sites of DNA DSBs and both bind to DNA). Understanding the architecture of Xrs2 could therefore offer critical insights into how NBS1 operates in humans. Here, we report the first high-resolution (2.38 Å) structure of the folded domains (FHA–BRCT1/2) of Xrs2 from *S. cerevisiae*, which will advance our understanding of its functions in the DNA damage response and telomere-length maintenance.

## 2. Materials and methods

### 2.1. Protein production and purification

#### 2.1.1. Cloning

The folded N-terminal region of *Sc* Xrs2 (Xrs2^325^; residues 1–325; UniProt P33301), based on the X-ray crystal structures of *Sp* Nbs1 (Williams *et al.*, 2009 ▸; Lloyd *et al.*, 2009 ▸), was subcloned from a yeast shuttle vector, generously provided by the Durocher Laboratory (Lunenfeld–Tanenbaum Research Institute, Toronto, Ontario, Canada), into the NdeI and NotI restriction sites of the pET-29 expression vector (Novagen). The reverse PCR primer included the sequence for a TEV protease-cleavable C-terminally 6×His-tagged protein, facilitating downstream purification.

The ligated plasmid was transformed into DH5α chemically competent *Escherichia coli* cells (Thermo Fisher Scientific) and plated on Luria–Bertani medium containing 50 µg ml^−1^ kanamycin (LB/Kan). Plasmid DNA was purified from single colonies using a Macherey–Nagel NucleoSpin Plasmid kit. The sequence was confirmed through Sanger sequencing (Genewiz). Details of the cloning procedure and protein expression are provided in Table 1 ▸.

#### 2.1.2. Protein expression and purification

The Xrs2^325^ expression plasmid was transformed into BL21 Star (DE3) chemically competent *E. coli* cells (Thermo Fisher Scientific) and plated on LB/Kan. The resulting colonies were cultured at 37°C in 750 ml LB/Kan. Protein expression was induced by adding 1 m*M* isopropyl β-d-1-thiogalactopyranoside (IPTG) when the culture reached an OD_600_ of between 0.8 and 1.0; Xrs2^325^ was expressed at 37°C for 4 h. Following expression, the cells were harvested by centrifugation at 4000*g* for 30 min, resuspended in lysis buffer (25 m*M* HEPES, 300 m*M* NaCl, 25 m*M* imidazole, 0.1% β-mercaptoethanol pH 7.0) and stored overnight at −20°C. The cells were thawed in an ice bath and incubated with 1 m*M* phenylmethylsulfonyl fluoride (PMSF) and 0.5 mg ml^−1^ lysozyme for 20 min with rocking on ice. Cell lysis was performed by homogenization (Avestin) and the lysate was cleared by centrifugation in a JA 25.50 rotor (Beckman Coulter) at 23 000 rev min^−1^ for 50 min at 4°C. The supernatant was filtered through a 0.45 µm syringe filter (Sartorius) and was loaded onto a 5 ml HisTrap HP column (Cytiva). The bound protein was washed with additional lysis buffer, then with lysis buffer plus 1.2 *M* NaCl, and was finally eluted with buffer *B* (25 m*M* HEPES, 300 m*M* NaCl, 300 m*M* imidazole, 0.1% β-mercaptoethanol pH 7.0). TEV protease (1:10 ODU ratio of TEV protease to the eluted protein) was added to cleave off the C-terminal 6×His-tag, and the sample was dialyzed overnight in 1 l fresh lysis buffer at 4°C. After dialysis, the sample was reloaded onto the 5 ml HisTrap HP column and the flowthrough was collected. The purified, cleaved Xrs2^325^ was concentrated using a 10 kDa molecular-weight cutoff centrifugal concentrator (Millipore), and further purification was performed using a HiLoad 16/600 Superdex 200 pg size-exclusion column (Cytiva) equilibrated with 25 m*M* HEPES, 300 m*M* NaCl, 0.1% β-mercaptoethanol pH 7. Fractions containing the ∼37 kDa Xrs2^325^ were pooled, buffer-exchanged into 100 m*M* NaCl, 25 m*M* HEPES, 1%(*v*/*v*) β-mercaptoethanol, 2 m*M* MgSO_4_ pH 7.0 and concentrated using a 10 kDa molecular-weight cutoff centrifugal concentrator.

### 2.2. Crystallization

Crystallization experiments were conducted using a protein solution containing Xrs2^325^ and a DNA hairpin in a 1:1 molar ratio to investigate the proposed interaction between the two macromolecules. The DNA (5′-CACGCACGTAGAAGCTTTTGCTTCTACGTGCGTGAC-3′) was ordered from Integrated DNA Technologies (IDT) and was buffer-exchanged into the protein–DNA buffer solution (Table 2 ▸). Hanging-drop crystallization was performed by mixing 2 µl of the protein–DNA solution with 2 µl well solution and allowing equilibration at room temperature for one week. Multiple crystallization plates were used to explore different conditions, and an optimal well condition of 3.0 *M* NaCl, 0.1 *M* Tris pH 8.3 was identified. Crystallization details are provided in Table 2 ▸.

### 2.3. Data collection and processing

Data were acquired on NSLS-II beamline 17-ID-2 with a Dectris EIGER 16M detector under 100 K cryo-conditions at a wavelength of 0.979 Å. Data were processed using *XDS* (Kabsch, 2010 ▸). Anisotropic diffraction analysis and truncation were performed with *STARANISO* (https://staraniso.globalphasing.org/). The X-ray diffraction data collection is summarized in Table 3 ▸.

### 2.4. Structure solution and structure refinement

Molecular replacement using the crystal structure of *Sp* Nbs1 (PDB entry 3hue; Williams *et al.*, 2009 ▸) did not provide adequate phasing information; therefore, molecular-replacement search models were generated via *AlphaFold*2 (Jumper *et al.*, 2021 ▸; Tunyasuvunakool *et al.*, 2021 ▸). Multiple *AlphaFold*2 models were generated and subsequently filtered based on their agreement with methyl-based solution-state NMR data (Vigneswaran *et al.*, 2025 ▸). The protein side chains of the best-fitting *AlphaFold*2 model were truncated using *CHAINSAW* (Stein, 2008 ▸) in *CCP*4 (Agirre *et al.*, 2023 ▸) and initial phases were obtained from *Phaser-MR*. Refinement was performed using *phenix.refine* (Liebschner *et al.*, 2019 ▸) and *Coot* (Emsley & Cowtan, 2004 ▸). After 67 cycles of refinement, *PDB-REDO* (Van Beusekom *et al.*, 2018 ▸; Joosten *et al.*, 2014 ▸) was implemented in an effort to improve the *R*_work_ and *R*_free_ values. Further refinement of the structure was carried out using *Phenix* and *Coot* for 46 further cycles to reduce the clashscore and the number of rotamer outliers, which led to the final resolved model with *R*_work_ and *R*_free_ values of 0.2982 and 0.3119, respectively. Details of the structure-refinement statistics are provided in Table 4 ▸. Note that no density was observed for the DNA molecule, and the final model is Xrs2^325^ alone. The final model has been deposited in the Research Collaboratory for Structural Bioinformatics Protein Data Bank as PDB entry 9ee7.

## 3. Results and discussion

Previous research has shown that full-length *Sc* Xrs2 binds to DNA (Trujillo *et al.*, 2003 ▸; Möller *et al.*, 2024 ▸). To structurally characterize this interaction, we attempted to co-crystallize the folded N-terminal region of *Sc* Xrs2 (residues 1–325) with DNA. A 9-, 11-, 13- or 15-base-pair hairpin DNA containing a four-nucleotide loop and a two-nucleotide 3′-overhang was added to *Sc* Xrs2^325^ at equimolar concentrations. Although the highest quality diffracting crystal was obtained for the 15-base-pair DNA hairpin and Xrs2^325^ complex, the final electron density showed no evidence of bound DNA. This result suggests that if the DNA does interact with the N-terminal region of *Sc* Xrs2, the interaction is too transient or unstable under the specific conditions used for crystal formation to be observed. Nevertheless, *Sc* Xrs2^325^ was successfully crystallized by the hanging-drop vapor-diffusion method at 25°C (Fig. 1 ▸*b*). The crystal belonged to space group *P*6_1_22, containing one Xrs2^325^ monomer in the asymmetric unit. The structure was determined at a resolution of 2.38 Å (Fig. 1 ▸*c*). Structural refinement yielded *R*_work_ and *R*_free_ values of 0.2982 and 0.3119, respectively. The loop residues Asn7–Gly13, Asn58–Leu63, Ser95–Val97 and Gly316–Lys325 were excluded from the structural model due to weak electron density in these regions.

Modeling the structure of Xrs2^325^ into the electron density was a difficult process. After 67 rounds of refinement with little significant improvement in the *R*_work_ and *R*_free_ values, *PDB-REDO* was utilized. *PDB-REDO* calculated a model with *R*_work_ and *R*_free_ values that were reduced from 0.2834 and 0.3073 to 0.2417 and 0.3015, respectively. However, the clashscore and the number of rotamer outliers increased. 46 additional rounds of refinement were used to reduce the clashscore and rotamer outliers, although this resulted in increased *R*_work_ and *R*_free_ values. Analysis of the geometric quality of the final model revealed strained backbone conformations in several loop regions: the Ramachandran statistics showed 1.03% and 11.30% of the residues in outlier and allowed space, respectively. Many of these residues map to disordered or flexible segments in the FHA and BRCT2 domains coinciding with omitted or poorly resolved loop regions. The difficulty in model building is further reflected in the elevated r.m.s.d. value for bond angles (1.12°).

**Figure 2:**
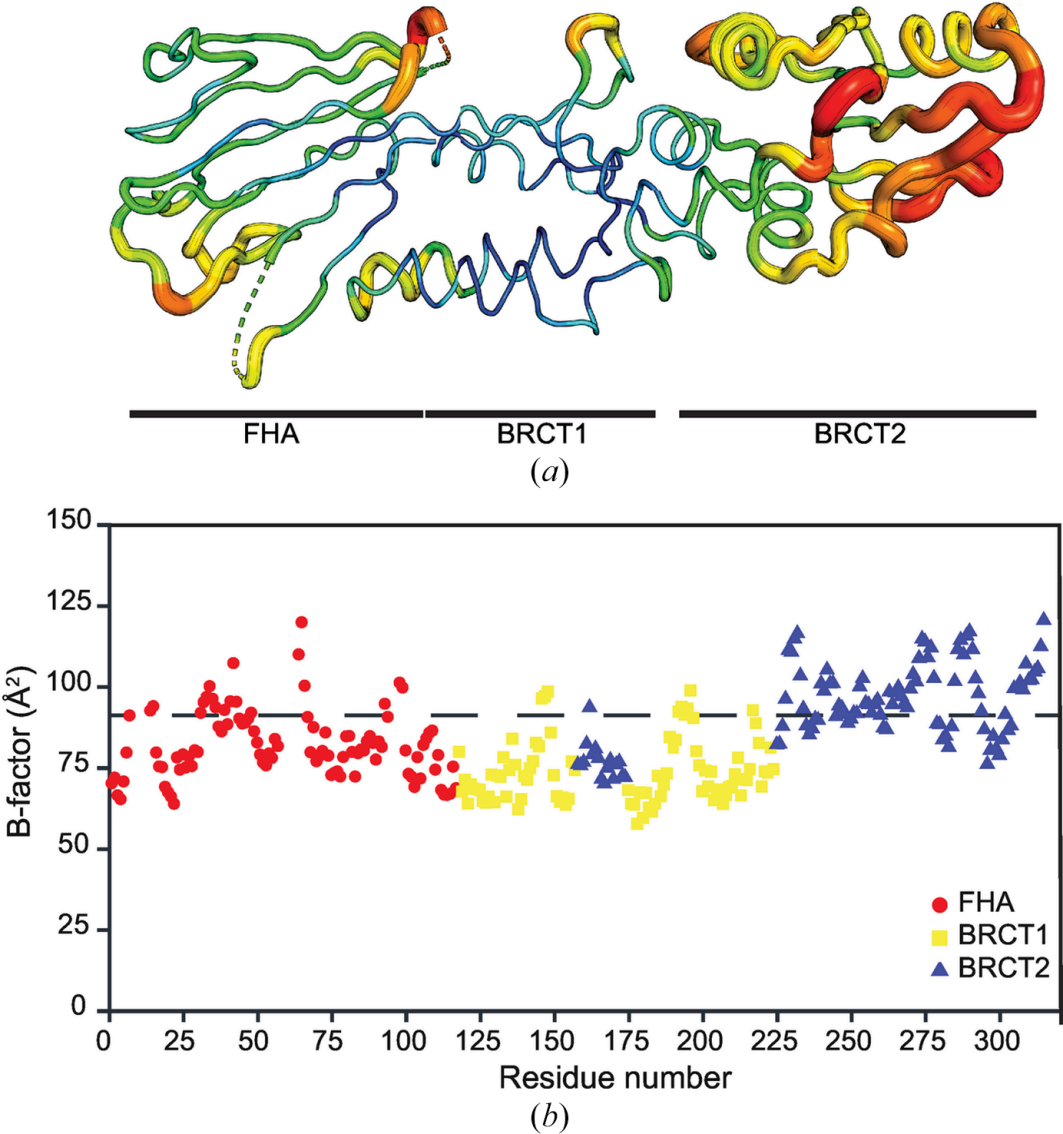
Analysis of Xrs2^325^ crystallographic *B* factors. (*a*) Putty representation of Xrs2^325^ illustrating crystallographic *B* factors. The tube thickness and blue–green–red color gradient depict backbone C^α^*B* factors. (*b*) Scatter plot of the average residue *B* factor versus residue number. Data points are colored by domain: FHA in red circles, BRCT1 in yellow squares and BRCT2 in blue triangles. The horizontal line depicts the global average *B* factor of 83.07 Å^2^.

To understand the reason for the difficulty in model building, we analyzed the calculated *B* factors (Fig. 2 ▸). The global average *B* factor for all atoms (excluding crystallo­graphic waters) in Xrs2^325^ was 83.70 Å^2^, with the smallest *B* factor in Lys178 of the BRCT1 domain (50.06 Å^2^) and the largest in His65 of the FHA domain (130.38 Å^2^). Visualization of the C^α^*B* factors on the structure (Fig. 2 ▸*a*) or the plot of the residue average *B* factor plotted against the sequence (Fig. 2 ▸*b*) reveals two phenomena. Firstly, BRCT1 has the smallest *B* factors (all-atom average of 72.85 Å^2^ for residues 118–157 and 175–224), whereas BRCT2 has the largest *B* factors (all-atom average of 92.84 Å^2^ for residues 158–174 and 225–315). The all-atom average *B* factor for the FHA domain is 81.08 Å^2^ (residues 1–117). This high level of disorder contrasts starkly with the previously published models of *Sp* Nbs1 (PDB entries 3hue and 3i0m), which have average *B* factors of 33.32 and 33.12 Å^2^, respectively. Secondly, within each domain many of the loop regions contain relatively high *B* factors. This trend is especially obvious for residues preceding/proceeding (for example, Asn7–Gly13) or adjacent to (Lys41–Asn42) omitted or poorly resolved loops, as expected (Djinovic-Carugo & Carugo, 2015 ▸). Additionally, the high solvent content (73.73%) of our crystal further increased the overall crystal mobility and disorder, contributing to the elevated *B* factors across the structure (Carugo, 2018 ▸). Together, the high level of disorder and highly flexible loop regions, particularly in the FHA domain, led to weak or missing electron density and made accurate modeling of side chains difficult without introducing steric clashes. In summary, the dynamic and/or disordered nature and structural strain within the FHA and BRCT2 loop regions posed a significant limitation for full atomic-level resolution.

**Figure 3:**
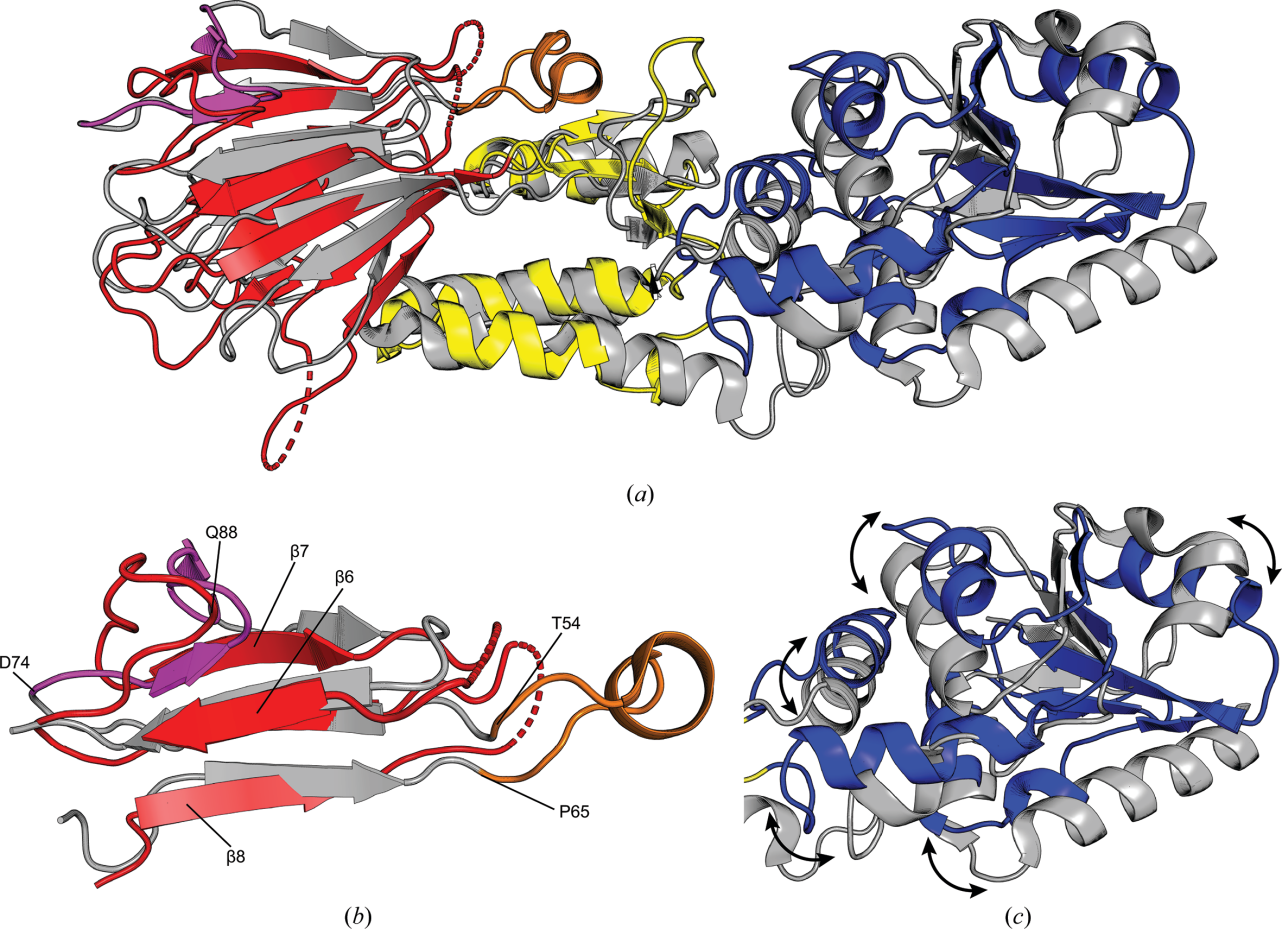
Structural comparison of *Sc* Xrs2^325^ and *Sp* Nbs1^329^. (*a*) Overlay of the crystal structure of *Sc* Xrs2^325^ (colored as in Fig. 1 ▸) with the crystal structure of *Sp* Nbs1^329^ (gray, PDB entry 3hue; Williams *et al.*, 2009 ▸). (*b*) Close-up view of the FHA region, highlighting differences in the loop region of *Sp* Nbs1 from The54 to Pro65 (depicted in orange) and Asp74 to Gln88 (magenta). (*c*) Isolated BRCT2 region, illustrating structural differences between the two proteins.

As seen in the previously published crystal structures of the homologous folded N-terminal region of *Sp* Nbs1 (Williams *et al.*, 2009 ▸; Lloyd *et al.*, 2009 ▸), the model of *Sc* Xrs2^325^ revealed an extended structure with approximate dimensions of 82 × 31 × 24 Å and three distinct domains organized sequentially from the N-terminus: FHA, BRCT1 and BRCT2 domains (Fig. 1 ▸*c*). The FHA domain is comprised of eight antiparallel β-strands, whereas the BRCT1 domain contains three α-helices and three short β-strands. The BRCT2 domain, in contrast, has six α-helices and five β-strands (Fig. 1 ▸*c*). A comparison of the *Sc* Xrs2^325^ crystal structure with the *Sp* Nbs1 structures revealed notable differences. Alignment with the crystal structure reported by Tainer and coworkers for *Sp* Nbs1 (PDB entry 3hue) resulted in a backbone-atom root-mean-squared deviation (r.m.s.d.) of 6.68 Å (*n* = 996 atoms), whereas the structure reported by Smerdon and coworkers for *Sp* Nbs1 (PDB entry 3i0m) had an r.m.s.d. of 6.32 Å (*n* = 1028 atoms) to the *Sc* Xrs2^325^ model (Fig. 3 ▸*a*). Generally, the overall structure of the FHA and BRCT1 domains in the three crystal structures is similar; however, several loops in the FHA domain showed potential differences between Xrs2 and Nbs1. Specifically, the unstructured loop between β_6_ and β_7_ in *Sc* Xrs2^325^ adopts a two-stranded β-sheet conformation in *Sp* Nbs1 (highlighted in magenta in Fig. 3 ▸*b*). Furthermore, the loop between β_5_ and β_6_ in *Sc* Xrs2 is unresolved, whereas the resolved loop in *Sp* Nbs1 contains an additional helix (highlighted in orange in Fig. 3 ▸*b*). In contrast, comparison of the BRCT2 domains revealed significant differences between *Sc* Xrs2 and *Sp* Nbs1 (Fig. 3 ▸*c*). Although the overall BRCT2 architecture is conserved, substantial variations were observed in the relative lengths of the α-helices and β-strands, and many of these secondary-structural elements are translated relative to one another. In support of this observation, the alignment of the FHA, BRCT1 and BRCT2 domains of *Sc* Xrs2^325^ with *Sp* Nbs1 (PDB entry 3hue) yielded backbone-atom r.m.s.d. values of 7.13 Å (*n* = 249 atoms), 8.09 Å (*n* = 108 atoms) and 10.61 Å (*n* = 177 atoms), respectively, indicating that the largest deviation occurs in the BRCT2 domain, consistent with the larger *B* factors observed for this domain. On the other hand, the *Sc* Xrs2 model in the *AlphaFold*2 database (version 4) gave a backbone-atom r.m.s.d. of 1.67 Å (*n* = 1196 atoms) relative to the crystal structure reported here. When compared with the *ColabFold* model of *Sc* Xrs2 that we previously filtered using residual dipolar couplings (Vigneswaran *et al.*, 2025 ▸), the r.m.s.d. was 1.61 Å (*n* = 1200 atoms). These r.m.s.d.s indicate similar backbone positioning between the computational models of *Sc* Xrs2, with much of the variation occurring in the side-chain positions.

**Figure 4:**
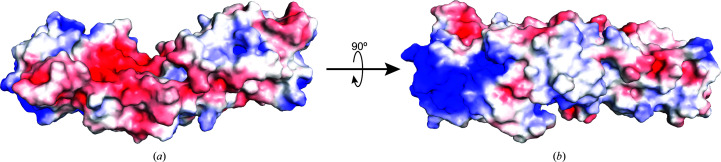
Electrostatic surface-charge representation of *Sc* Xrs2^325^. (*a*) The electrostatic surface potential was calculated using *APBS* and is colored according to calculated charge from red (−5 *kT*/e) to blue (+5 *kT*/e). (*b*) The structure in (*a*) rotated 90°.

To explore the potential bipartite binding framework, we analyzed the surface-charge distribution of Xrs2^325^. The *Sc* Xrs2^325^ crystal structure lacks several side chains, which prevented electrostatic surface analysis using *Adaptive Poisson–Boltzmann Solver* (*APBS*) electrostatics calculations. To address this, the side-chain heavy atoms which were not modeled were reintroduced to the calculated structure using *Phenix* and *Coot*, and the electrostatic surface potential was determined. The surface-charge distribution revealed distinct positively and negatively charged regions, which may play a role in protein–protein or protein–DNA interactions (Fig. 4 ▸). A prominent positively charged surface is localized on the FHA domain and is formed by residues Arg32, Lys35, Lys41, Lys44, Arg48, His50, Lys54, Lys73, Lys75, Lys81, Lys82 and Lys85 (Fig. 4 ▸). A similar positively charged surface was observed in the X-ray crystal structures of *Sp* Nbs1 and forms the binding site for the phosphothreonine peptide derived from Ctp1 (Williams *et al.*, 2009 ▸). Conservation of this charged region suggests that the homologous phosphorylated *Sc* Sae2 peptide binds to the same region. Thus, this region serves as a key site for binding partners, accommodating post-translationally modified proteins, such as those containing phosphorylated serine or phosphorylated threonine, or potentially other negatively charged biomolecules such as DNA.

**Figure 5:**
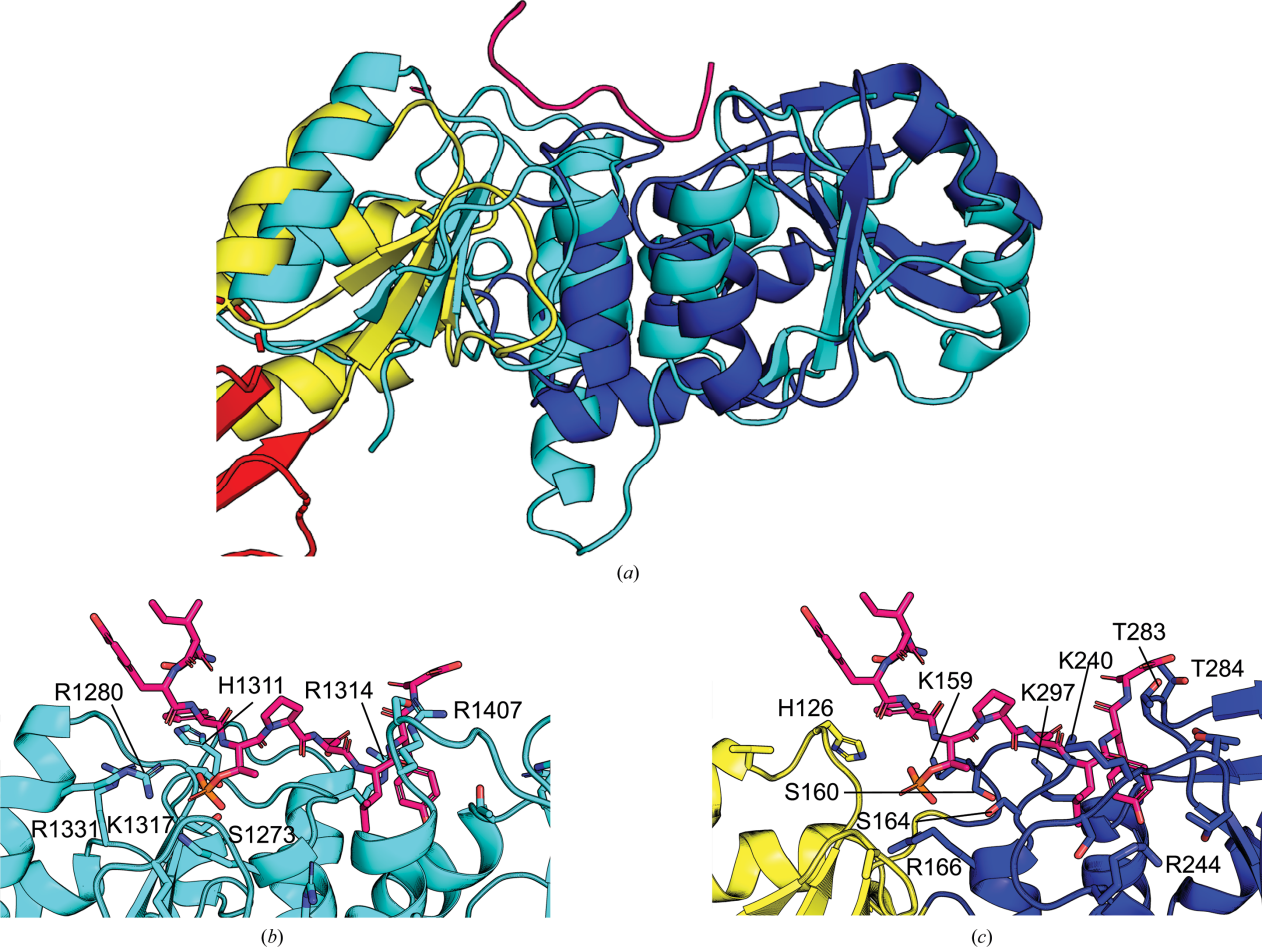
Structural comparison of *Sc* Xrs2 and the TopBP1 BRCT7/8–BACH1 complex. (*a*) Structural overlay of the crystal structure of *Sc* Xrs2^325^ (colored as in Fig. 1 ▸) with the TopBP1 BRCT7/8–BACH1 complex (teal and magenta; PDB entry 3al3; Leung *et al.*, 2011 ▸). (*b*) Detailed view of the BRCT 7/8 region of TopBP1 interacting with BACH1. (*c*) Detailed view of the BRCT1/2 region (yellow and blue) of *Sc* Xrs2^325^, with the BACH1 peptide (red) aligned to highlight the presence of numerous charged residues in Xrs2, suggesting a potential binding site for protein interactions.

To explore potential ligand binding in the BRCT repeats of *Sc* Xrs2, we compared our structure with that of the human TopBP1 tandem BRCT7/8 domains (PDB entry 3al3; Leung *et al.*, 2011 ▸), which has the BACH1 phosphopeptide bound to a positively charged region between its tandem BRCT domains. An overlay of Xrs2^325^ with the TopBP1 BRCT7/8 domains revealed a similar, moderately positively charged patch in the BRCT1/2 domains of Xrs2^325^, formed by residues His126, Arg131, Lys159, Arg166, Lys240 and Lys244 (Fig. 5 ▸). This suggests that the BRCT1/2 domain of Xrs2^325^ could bind negatively charged partners in a similar manner.

Interestingly, a negatively charged region, located between the FHA and BRCT1 domains and formed by residues Glu113, Glu115, Glu119, Asp201 and Glu202 (Fig. 4 ▸), is positioned 180° opposite to the positively charged patch in BRCT1/2. This negatively charged region is also conserved in the BRCT domains of *Sp* Nbs1 and TopBP1. Thus, this region could play a role in directing positively charged partners to bind either to the FHA domain or within BRCT1/2. Alternatively, it raises the possibility that this negatively charged region itself could interact with a positively charged partner.

**Figure 6:**
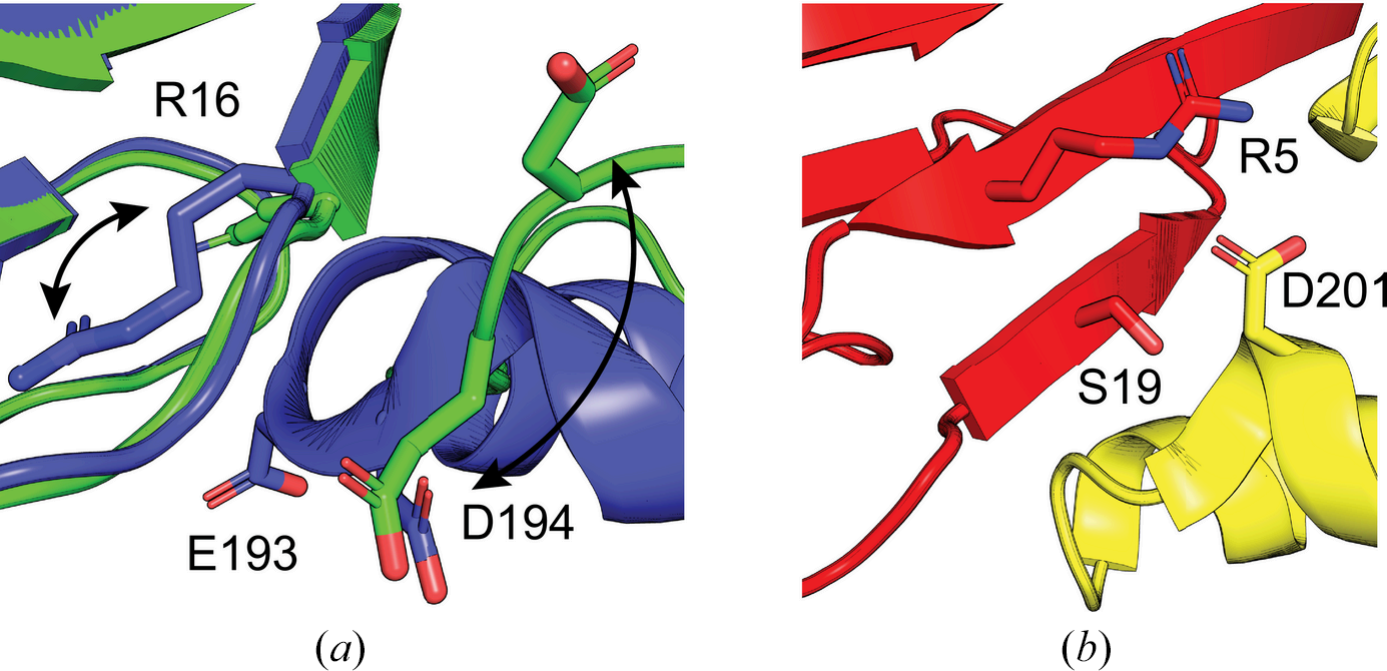
Structural interface between the FHA and BRCT1 domains of *Sp* Nbs1 and *Sc* Xrs2, highlighting structural analogy. (*a*) Structural overlay of *Sp* Nbs1 in its Ctp1-bound (PDB entry 3huf, green) and apo (PDB entry 3hue, blue) states (Williams *et al.*, 2009 ▸). Upon Ctp1 binding, residue Arg16 within the FHA domain undergoes a conformational flip, acting as a molecular switch that disrupts its interaction with BRCT1-domain residues Glu193 and Asp194. (*b*) In *Sc* Xrs2, FHA-domain residues Arg5 and Ser19 are positioned in proximity to BRCT1-domain residue Asp201, forming an interface structurally analogous to that observed in *Sp* Nbs1 in (*a*).

A previous study by Tainer and coworkers proposed an arginine ‘switch’ (Arg16) in the FHA domain of *Sp* Nbs1, which rotates upon Ctp1 binding to interact with Glu193 and Asp194 in the BRCT1 domain (Fig. 6 ▸*a*). This switch may trigger an allosteric movement of the BRCT2 domain, resulting in a BRCT2 domain rotation of ∼20°. Intriguingly, the crystal structure of *Sc* Xrs2^325^ revealed structurally analogous residues in the FHA domain: Arg5 and Ser19 in the FHA domain are positioned near Asp201 in the BRCT1 domain and may mirror the role of the *Sp* Nbs1 arginine switch (Fig. 6 ▸*b*). Multiple sequence alignment across various species (Becker *et al.*, 2006 ▸) demonstrated that these charged residues are conserved at equivalent positions, highlighting the evolutionary preservation of this regulatory mechanism.

## 4. Conclusion

This study is the first experimental structural elucidation of the N-terminal folded core of *Sc* Xrs2 and provides interesting insights into its molecular architecture. Firstly, it confirms the presence of the conserved FHA–BRCT1–BRCT2 domains in this protein. Secondly, analysis of the *B* factors suggests significant disorder and/or dynamics within the loop regions of the FHA domain and the entire BRCT2 domain. Additionally, comparative analyses with homologous structures demonstrate the presence of a highly conserved positively charged patch within the FHA domain and tandem BRCT1/2 domains, alongside a moderately conserved negatively charged patch situated between the FHA and BRCT1 domains. These conserved electrostatic features play an important role in mediating the protein–protein interactions essential for the repair of damaged DNA.

## Supplementary Material

PDB reference: folded domains of Xrs2 from *S. cerevisiae*, 9ee7

## Figures and Tables

**Table 1:** Cloning and protein-expression information

Source organism	*S. cerevisiae*
DNA source	Yeast shuttle vector (gift from Durocher Laboratory)
Forward primer	5′-GATCCATATGTGGGTAGTACGATACCAGAAT-3′
Reverse primer	5′-GATCGCGGCCGCTCAATGGTGATGATGGTGGTGCCCAGCGGATTGGAAGTACAGGTTCTCACCGTTATCTAGAGTTCTTG-3′
Plasmid construction method	Restriction–ligation
Expression vector	pET-29
Expression host	*E. coli* BL21 Star (DE3)
Protein sequence	MWVVRYQNTLEDGSISFISCCLQAFKTYSIGRSSKNPLIIKNDKSISRQHITFKWEINNSSDLKHSSLCLVNKGKLTSLNKKFMKVGETFTINASDVLKSTIIELGTTPIRIEFEWINEVWNIPPHLTQFRTMLSEYGISTEISINDIPANLMISDYPKSEDNSIRELYALVSTIPMKKSRFLMELCNTLLPTSKTNLKFDEMWNDMISNPEYNVFDFDPNILLSKFMRLNNIRVLTTIKSEPRLSSLLRTFNINLFAFDNIDSLYKYVDSLEASTEYLILTTTDKKENGKILCTIKTMLTSIIDGTLSAVINMKGASSRTLDNGENLYFQ

**Table 2:** Crystallization conditions

Method	Hanging-drop vapor diffusion
Plate type	24-well plate, Hampton Research
Temperature (K)	298
Protein concentration (mg ml^−1^)	5
DNA concentration (mg ml^−1^)	1.6
Protein/DNA buffer solution	100 m*M* NaCl, 25 m*M* HEPES, 1%(*v*/*v*) β-mercaptoethanol, 2 m*M* MgSO_4_ pH 7.0
Volume and ratio of drop	4 µl, 2:2 (protein:reservoir)
Volume of reservoir (µl)	500

**Table 3:** X-ray data collection Values in parentheses are for the outer shell.

X-ray source	NSLS-II beamline 17-ID-2
Wavelength (Å)	0.97933
Data-collection temperature (K)	100
Detector	Dectris EIGER 16M
Exposure time per frame (s)	0.02
Crystal-to-detector distance (mm)	200
Angle increment (°)	0.20
Resolution range (Å)	71.58–2.38 (2.43–2.38)†
Space group	*P*6_1_22
*a*, *b*, *c* (Å)	84.84, 84.84, 316.71
α, β, γ (°)	90, 90, 120
Matthews coefficient (Å^3^ Da^−1^)	4.43
Solvent content (%)	72.21
Total reflections	631578
Unique reflections	27755 (1350)
Multiplicity	21.7
Mosaicity (°)	0.2
Completeness (%)	99.8 (31.88)
Mean *I*/σ(*I*)	16.4 (1.1)
Wilson *B* factor (Å^2^)	74.10
*R*_merge_	0.141 (3.61)
*R*_meas_	0.144 (3.70)
*R*_p.i.m._	0.031 (0.82)
CC_1/2_	0.999 (0.444)

† Ellipsoidal 2.943 Å 0.894a* − 0.447b*, 2.943 Å b*, 2.174 Å c*.

**Table 4:** Structure-refinement statistics

Reflections used in refinement	25538
Reflections used for *R*_free_	1284
*R*_work_	0.2982
*R*_free_	0.3119
No. of non-H atoms	
Total	2183
Macromolecules	2176
Ligands	—
Solvent	7
No. of protein residues	300
R.m.s.d., bond lengths (Å)	0.009
R.m.s.d., angles	1.12
Ramachandran favored (%)	87.67
Ramachandran allowed (%)	11.30
Ramachandran outliers (%)	1.03

## References

[bb1] Agirre, J., Atanasova, M., Bagdonas, H., Ballard, C. B., Baslé, A., Beilsten-Edmands, J., Borges, R. J., Brown, D. G., Burgos-Mármol, J. J., Berrisford, J. M., Bond, P. S., Caballero, I., Catapano, L., Chojnowski, G., Cook, A. G., Cowtan, K. D., Croll, T. I., Debreczeni, J. É., Devenish, N. E., Dodson, E. J., Drevon, T. R., Emsley, P., Evans, G., Evans, P. R., Fando, M., Foadi, J., Fuentes-Montero, L., Garman, E. F., Gerstel, M., Gildea, R. J., Hatti, K., Hekkelman, M. L., Heuser, P., Hoh, S. W., Hough, M. A., Jenkins, H. T., Jiménez, E., Joosten, R. P., Keegan, R. M., Keep, N., Krissinel, E. B., Kolenko, P., Kovalevskiy, O., Lamzin, V. S., Lawson, D. M., Lebedev, A. A., Leslie, A. G. W., Lohkamp, B., Long, F., Malý, M., McCoy, A. J., McNicholas, S. J., Medina, A., Millán, C., Murray, J. W., Murshudov, G. N., Nicholls, R. A., Noble, M. E. M., Oeffner, R., Pannu, N. S., Parkhurst, J. M., Pearce, N., Pereira, J., Perrakis, A., Powell, H. R., Read, R. J., Rigden, D. J., Rochira, W., Sammito, M., Sánchez Rodríguez, F., Sheldrick, G. M., Shelley, K. L., Simkovic, F., Simpkin, A. J., Skubak, P., Sobolev, E., Steiner, R. A., Stevenson, K., Tews, I., Thomas, J. M. H., Thorn, A., Valls, J. T., Uski, V., Usón, I., Vagin, A., Velankar, S., Vollmar, M., Walden, H., Waterman, D., Wilson, K. S., Winn, M. D., Winter, G., Wojdyr, M. & Yamashita, K. (2023). *Acta Cryst.* D**79**, 449–461.

[bb2] Becker, E., Meyer, V., Madaoui, H. & Guerois, R. (2006). *Bioinformatics*, **22**, 1289–1292. 10.1093/bioinformatics/btl075 16522671

[bb3] Bunting, S. F. & Nussenzweig, A. (2013). *Nat. Rev. Cancer*, **13**, 443–454. 10.1038/nrc3537 PMC5724777 23760025

[bb4] Carney, J. P., Maser, R. S., Olivares, H., Davis, E. M., Le Beau, M., Yates, J. R., Hays, L., Morgan, W. F. & Petrini, J. H. J. (1998). *Cell*, **93**, 477–486. 10.1016/s0092-8674(00)81175-7 9590181

[bb5] Carugo, O. (2018). *BMC Bioinformatics*, **19**, 61. 10.1186/s12859-018-2083-8 PMC5824579 29471780

[bb6] Chrzanowska, K. H., Gregorek, H., Dembowska-Bagińska, B., Kalina, M. A. & Digweed, M. (2012). *Orphanet J. Rare Dis.* **7**, 13. 10.1186/1750-1172-7-13 PMC3314554 22373003

[bb7] Djinovic-Carugo, K. & Carugo, O. (2015). *Intrinsically Disord. Proteins*, **3**, e1095697. 10.1080/21690707.2015.1095697 PMC5314880 28232893

[bb8] Durocher, D., Henckel, J., Fersht, A. R. & Jackson, S. P. (1999). *Mol. Cell*, **4**, 387–394. 10.1016/s1097-2765(00)80340-8 10518219

[bb9] Emsley, P. & Cowtan, K. (2004). *Acta Cryst.* D**60**, 2126–2132. 10.1107/S0907444904019158 15572765

[bb10] Han, J. & Huang, J. (2020). *Genome Instab. Dis.* **1**, 10–19.

[bb11] Heikkinen, K. (2005). *Carcinogenesis*, **27**, 1593–1599. 10.1093/carcin/bgi360 PMC3006189 16474176

[bb12] Joosten, R. P., Long, F., Murshudov, G. N. & Perrakis, A. (2014). *IUCrJ*, **1**, 213–220. 10.1107/S2052252514009324 PMC4107921 25075342

[bb13] Jumper, J., Evans, R., Pritzel, A., Green, T., Figurnov, M., Ronneberger, O., Tunyasuvunakool, K., Bates, R., Žídek, A., Potapenko, A., Bridgland, A., Meyer, C., Kohl, S. A. A., Ballard, A. J., Cowie, A., Romera-Paredes, B., Nikolov, S., Jain, R., Adler, J., Back, T., Petersen, S., Reiman, D., Clancy, E., Zielinski, M., Steinegger, M., Pacholska, M., Berghammer, T., Bodenstein, S., Silver, D., Vinyals, O., Senior, A. W., Kavukcuoglu, K., Kohli, P. & Hassabis, D. (2021). *Nature*, **596**, 583–589. 10.1038/s41586-021-03819-2 PMC8371605 34265844

[bb14] Kabsch, W. (2010). *Acta Cryst.* D**66**, 125–132. 10.1107/S0907444909047337 PMC2815665 20124692

[bb15] Kim, J.-E., Minter-Dykhouse, K. & Chen, J. (2006). *Mol. Carcinog.* **45**, 403–408. 10.1002/mc.20221 16691596

[bb16] Kim, J. H., Grosbart, M., Anand, R., Wyman, C., Cejka, P. & Petrini, J. H. J. (2017). *Cell. Rep.* **18**, 496–507. 10.1016/j.celrep.2016.12.035 PMC5234850 28076792

[bb17] Kobayashi, J., Tauchi, H., Sakamoto, S., Nakamura, A., Morishima, K., Matsuura, S., Kobayashi, T., Tamai, K., Tanimoto, K. & Komatsu, K. (2002). *Curr. Biol.* **12**, 1846–1851. 10.1016/s0960-9822(02)01259-9 12419185

[bb18] Lamarche, B. J., Orazio, N. I. & Weitzman, M. D. (2010). *FEBS Lett.* **584**, 3682–3695. 10.1016/j.febslet.2010.07.029 PMC2946096 20655309

[bb19] Lengauer, C., Kinzler, K. W. & Vogelstein, B. (1998). *Nature*, **396**, 643–649. 10.1038/25292 9872311

[bb20] Leung, C. C. Y., Gong, Z., Chen, J. & Glover, J. N. M. (2011). *J. Biol. Chem.* **286**, 4292–4301. 10.1074/jbc.M110.189555 PMC3039391 21127055

[bb21] Lieber, M. R. (2010). *Annu. Rev. Biochem.* **79**, 181–211. 10.1146/annurev.biochem.052308.093131 PMC3079308 20192759

[bb22] Liebschner, D., Afonine, P. V., Baker, M. L., Bunkóczi, G., Chen, V. B., Croll, T. I., Hintze, B., Hung, L.-W., Jain, S., McCoy, A. J., Moriarty, N. W., Oeffner, R. D., Poon, B. K., Prisant, M. G., Read, R. J., Richardson, J. S., Richardson, D. C., Sammito, M. D., Sobolev, O. V., Stockwell, D. H., Terwilliger, T. C., Urzhumtsev, A. G., Videau, L. L., Williams, C. J. & Adams, P. D. (2019). *Acta Cryst.* D**75**, 861–877. 10.1107/S2059798319011471 PMC6778852 31588918

[bb23] Lloyd, J., Chapman, J. R., Clapperton, J. A., Haire, L. F., Hartsuiker, E., Li, J., Carr, A. M., Jackson, S. P. & Smerdon, S. J. (2009). *Cell*, **139**, 100–111. 10.1016/j.cell.2009.07.043 PMC2900601 19804756

[bb24] Lu, C.-S., Truong, L. N., Aslanian, A., Shi, L. Z., Li, Y., Hwang, P. Y.-H., Koh, K. H., Hunter, T., Yates, J. R., Berns, M. W. & Wu, X. (2012). *J. Biol. Chem.* **287**, 43984–43994. 10.1074/jbc.M112.421545 PMC3527981 23115235

[bb25] Möller, C., Sharma, R., Öz, R., Reginato, G., Cannavo, E., Ceppi, I., Sriram, K. K., Cejka, P. & Westerlund, F. (2024). *Biochem. Biophys. Res. Commun.* **695**, 149464. 10.1016/j.bbrc.2023.149464 38217957

[bb26] Oh, J., Al-Zain, A., Cannavo, E., Cejka, P. & Symington, L. S. (2016). *Mol. Cell*, **64**, 405–415. 10.1016/j.molcel.2016.09.011 PMC5123801 27746018

[bb27] Rahman, S., Canny, M. D., Buschmann, T. A. & Latham, M. P. (2020). *Cells*, **9**, 1678. 10.3390/cells9071678 PMC7407228 32668560

[bb28] Romero-Laorden, N. & Castro, E. (2017). *Curr. Probl. Cancer*, **41**, 251–264. 10.1016/j.currproblcancer.2017.02.009 28454847

[bb29] Schiller, C. B., Lammens, K., Guerini, I., Coordes, B., Feldmann, H., Schlauderer, F., Möckel, C., Schele, A., Strässer, K., Jackson, S. P. & Hopfner, K.-P. (2012). *Nat. Struct. Mol. Biol.* **19**, 693–700. 10.1038/nsmb.2323 PMC3392456 22705791

[bb30] Seemanová, E., Sperling, K., Neitzel, H., Varon, R., Hadac, J., Butova, O., Schröck, E., Seeman, P. & Digweed, M. (2006). *J. Med. Genet.* **43**, 218–224. 10.1136/jmg.2005.035287 PMC2563240 16033915

[bb31] Stein, N. (2008). *J. Appl. Cryst.* **41**, 641–643.

[bb32] Trujillo, K. M., Roh, D. H., Chen, L., Van Komen, S., Tomkinson, A. & Sung, P. (2003). *J. Biol. Chem.* **278**, 48957–48964. 10.1074/jbc.M309877200 14522986

[bb33] Tunyasuvunakool, K., Adler, J., Wu, Z., Green, T., Zielinski, M., Žídek, A., Bridgland, A., Cowie, A., Meyer, C., Laydon, A., Velankar, S., Kleywegt, G. J., Bateman, A., Evans, R., Pritzel, A., Figurnov, M., Ronneberger, O., Bates, R., Kohl, S. A. A., Potapenko, A., Ballard, A. J., Romera-Paredes, B., Nikolov, S., Jain, R., Clancy, E., Reiman, D., Petersen, S., Senior, A. W., Kavukcuoglu, K., Birney, E., Kohli, P., Jumper, J. & Hassabis, D. (2021). *Nature*, **596**, 590–596. 10.1038/s41586-021-03828-1 PMC8387240 34293799

[bb34] van Beusekom, B., Touw, W. G., Tatineni, M., Somani, S., Rajagopal, G., Luo, J., Gilliland, G. L., Perrakis, A. & Joosten, R. P. (2018). *Protein Sci.* **27**, 798–808. 10.1002/pro.3353 PMC5818736 29168245

[bb35] Vigneswaran, A., Buschmann, T. A. & Latham, M. P. (2025). *J. Magn. Reson.* **374**, 107865. 10.1016/j.jmr.2025.107865 PMC11993329 40058108

[bb36] Wang, Z., Cummins, J. M., Shen, D., Cahill, D. P., Jallepalli, P. V., Wang, T.-L., Parsons, D. W., Traverso, G., Awad, M., Silliman, N., Ptak, J., Szabo, S., Willson, J. K. V., Markowitz, S. D., Goldberg, M. L., Karess, R., Kinzler, K. W., Vogelstein, B., Velculescu, V. E. & Lengauer, C. (2004). *Cancer Res.* **64**, 2998–3001. 10.1158/0008-5472.can-04-0587 15126332

[bb37] Williams, R. S., Dodson, G. E., Limbo, O., Yamada, Y., Williams, J. S., Guenther, G., Classen, S., Glover, J. N. M., Iwasaki, H., Russell, P. & Tainer, J. A. (2009). *Cell*, **139**, 87–99. 10.1016/j.cell.2009.07.033 PMC2762657 19804755

[bb38] Wu, H.-H., Wu, P.-Y., Huang, K.-F., Kao, Y.-Y. & Tsai, M.-D. (2012). *Biochemistry*, **51**, 575–577. 10.1021/bi201709w 22211259

[bb39] Wu, X., Ranganathan, V., Weisman, D. S., Heine, W. F., Ciccone, D. N., O’Neill, T. B., Crick, K. E., Pierce, K. A., Lane, W. S., Rathbun, G., Livingston, D. M. & Weaver, D. T. (2000). *Nature*, **405**, 477–482. 10.1038/35013089 10839545

